# Dynamic modeling of ABA-dependent expression of the *Arabidopsis RD29A* gene

**DOI:** 10.3389/fpls.2022.928718

**Published:** 2022-08-26

**Authors:** Ruth Ndathe, Renee Dale, Naohiro Kato

**Affiliations:** ^1^Department of Biological Sciences, Louisiana State University, Baton Rouge, LA, United States; ^2^Donald Danforth Plant Science Center, St. Louis, MO, United States

**Keywords:** ODEs, ABA, gene regulatory network, ABRE, DRE, RD29A, mathematical model, feedback regulation

## Abstract

The abscisic acid (ABA) signaling pathway is the key defense mechanism against drought stress in plants. In the pathway, signal transduction among four core proteins, pyrabactin resistance (PYR), protein phosphatase 2C (PP2C), sucrose-non-fermenting-1-related protein kinase 2 (SnRK2), and ABRE binding factor (ABF) leads to altered gene expression kinetics that is driven by an ABA-responsive element (ABRE). A most recent and comprehensive study provided data suggesting that ABA alters the expression kinetics in over 6,500 genes through the ABF-ABRE associations in *Arabidopsis*. Of these genes, termed ABA gene regulatory network (GRN), over 50% contain a single ABRE within 4 kb of the gene body, despite previous findings suggesting that a single copy of ABRE is not sufficient to drive the gene expression. To understand the expression system of the ABA GRN by the single ABRE, a dynamic model of the gene expression for the desiccation 29A (*RD29A*) gene was constructed with ordinary differential equations. Parameter values of molecular-molecular interactions and enzymatic reactions in the model were implemented from the data obtained by previously conducted *in vitro* experiments. On the other hand, parameter values of gene expression and translation were determined by comparing the kinetics of gene expression in the model to the expression kinetics of *RD29A* in real plants. The optimized model recapitulated the trend of gene expression kinetics of *RD29A* in ABA dose–response that were previously investigated. Further analysis of the model suggested that a single ABRE controls the time scale and dynamic range of the ABA-dependent gene expression through the PP2C feedback regulation even though an additional *cis*-element is required to drive the expression. The model construed in this study underpins the importance of a single ABRE in the ABA GRN.

## Introduction

Plants possess defense mechanisms against abiotic stresses (Basu et al., 2016; Kumar et al., 2018; Takahashi et al., 2020). One of the primary mechanisms is the abscisic acid (ABA) signaling pathway. ABA is a phytohormone that is produced under abiotic stresses such as drought conditions (Zeevaart and Creelman, 1988; Sauter et al., 2001; Ikegami et al., 2008). The ABA signaling pathway has been well-characterized, leading to downstream ABA responses such as stomatal closure and gene expression that help the plant acquire drought stress resistance (Steuer et al., 1988; Fujii et al., 2009; Umezawa et al., 2009). The most upstream of the core components in the ABA signaling pathway are ABA receptors named pyrabactin resistance/pyr1-like/ regulatory components of ABA receptors (PYR/PYL/RCAR) that bind ABA and, in turn, interact with different protein phosphatase 2Cs (PP2Cs), namely aba insensitive1/2 (ABI1/ABI2), hypersensitive to aba1/2 (HAB1/HAB2), aba-hypersensitive germination 3 (AHG3/PP2CA), and highly aba induced 1/2/3 (HA1/2/3). The PP2Cs inhibit SNF1-related protein kinase 2 s (SnRK2s), including SnRK2.2, SnRK2.3, and SnRK2.6, when they are not interacting with PYR (Rodriguez et al., 1998; Gosti et al., 1999; Merlot et al., 2001; Saez et al., 2004; Ma et al., 2009; Melcher et al., 2009; Nishimura et al., 2009; Park et al., 2009; Santiago et al., 2009; Yin et al., 2009; Soon et al., 2012). For gene expression, activated SnRK2s phosphorylate ABA-responsive elements (ABRE) binding factors 1/2/3/4 (ABF1/2/3/4). These phosphorylated transcription factors bind ABRE, a regulatory region of ABA-induced genes (Choi et al., 2000; Uno et al., 2000; Yoshida et al., 2015). For stomatal closure, the activated SnRK2, namely SnRK2.6 kinase, phosphorylate the slow-anion channels (SLAC1), leading to an anion and K^+^ efflux and eventual solute loss from the guard cells (Schroeder et al., 1984; Geiger et al., 2009; Lee et al., 2009; Albert et al., 2017).

A relationship between ABF-ABRE associations and kinetics of ABA-dependent gene expression has been studied extensively to understand the downstream response that changes growth and physiology with a function of time in plants. The current understanding is that a single copy of ABRE is not sufficient for the ABA-dependent expression (Shen et al., 1996; Hobo et al., 1999). Genes containing several ABREs in the promoter are mainly regulated by the ABA signaling pathway (Yamaguchi-Shinozaki and Shinozaki, 1994; Fujii et al., 2009; Ma et al., 2009). Namely, a pair of ABREs has been shown to be overrepresented in the promoter region of ABA-inducible genes in *Arabidopsis* (Zhang et al., 2005; Gómez-Porras et al., 2007). However, the data obtained in the most recent study with DAP-Seq (DNA affinity purification and sequencing) and RNA-seq (RNA sequencing) suggested that over 50% of the genes regulated by the ABF-ABRE associations contain a single ABRE within 4 kb of the gene body (Sun et al., 2022). This new data set suggests that genes containing a single ABRE are the primary target of the ABA signaling pathway. Although the requirement of the second *cis*-element for the expression of these genes has been shown (Narusaka et al., 2003), how the ABRE and second *cis*-elements co-operatively change the kinetics of ABA-dependent gene expression is little understood.

Dynamic modeling is a powerful tool that integrates extensive experimental data of pathway components, improving our understanding of the signaling pathway dynamics and making novel hypotheses and predictions (Poolman et al., 2004; Aldridge et al., 2006; Janes and Yaffe, 2006; Thakar et al., 2007). The network connectivity of the core components of the ABA signaling pathway has been revealed. Furthermore, *in vitro* parameters for many of the interactions of the core components have been experimentally determined, making this a good candidate for modeling.

This study aims to build a dynamic model of ABA-responsive gene expression with a single ABRE element, namely the *RD29A* gene that has been used as the marker for the ABA signaling pathway in *Arabidopsis*. The *RD29A* gene requires DRE (dehydration responsive element) to which DREB2A (DRE binding protein 2A) binds (Yamaguchi-Shinozaki and Shinozaki, 1994; Liu et al., 1998) in addition to ABRE for its expression (Yamaguchi-Shinozaki et al., 1995; Narusaka et al., 2003). Because the network structure of the *RD29A* gene was well established and the parameter values were already obtained experimentally, testing the effects of network structures on the model performance was not focused on in this study. Approximate curve fitting of the model output to actual plant data was conducted by optimizing parameter values of transcription and translation, which were not determined previously. This report describes how we built, optimized, and validated the model. The resulting model led us to form a new hypothesis that a single ABRE is not sufficient to derive the gene expression yet controls the time scale and the dynamic range of the ABA-dependent gene expression kinetics.

## Description

### Identification of ABA-induced genes containing a single ABRE in *Arabidopsis*

Supplementary Data 2 in the publication by Sun et al. (2022) was used to extract the data on genes that are differentially expressed by ABA and the number of ABRE in the genes in *Arabidopsis*. In the publication, gene expression was determined with RNA-seq. DEG (differentially expressed gene) was defined by DESeq2 as a gene whose *value of p* for the significance of differential gene expression is less than 0.05 in 10 μM ABA-treated samples compared to controls. Seven days old seedlings (shoot and root tissues) and two-time points (3 h and 24 h after the treatment) were compared. The number of ABREs in a gene was determined with DAP-seq. The ABRE element was defined as an ACGT sequence onto which ABF1, 2, 3, or 4 binds within the 5’ 2Kb upstream and 3’ 2Kb downstream in a gene’s protein-coding sequence (gene body).

### Construction of the dynamic model of the *RD29A* gene expression

**Figure 1:**
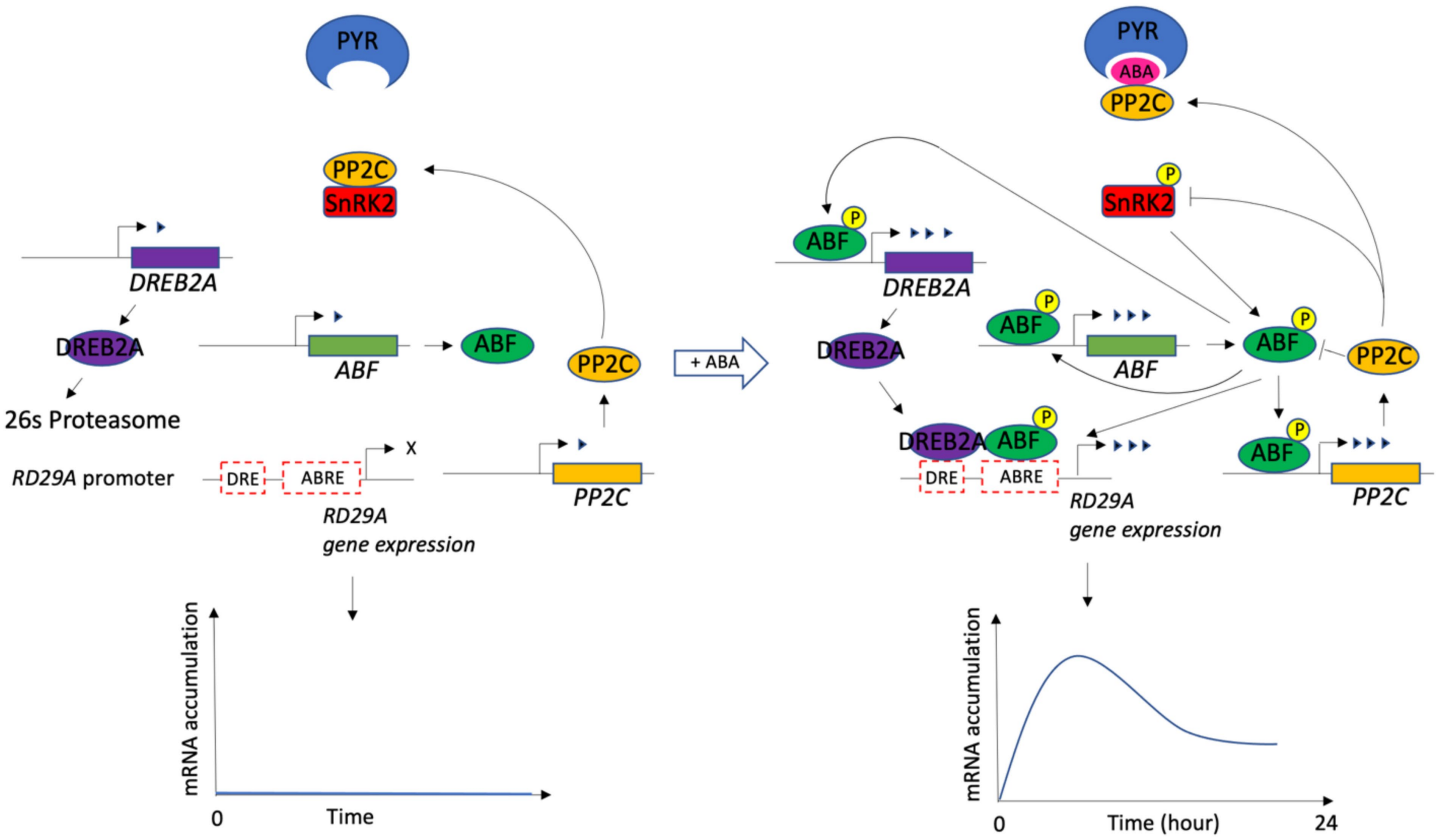
Schematic drawing of the ABA signaling pathway for the *RD29A* expression. ABA signaling core components and their interactions are shown with and without ABA. In the presence of ABA, downstream regulators ABF and DREB2A bind ABRE and DRE, the *cis*-elements on the RD29A gene, leading to its transient expression. The drawing was modified from the figure in (Wang et al., 2019). P with a circle represents the phosphorylation of a protein. A different number of arrowheads indicates a different level of gene expression. X indicates no gene expression.

A previous study defined a minimal set of core components and the signal transduction that led to ABA-induced gene expression (Yoshida et al., 2014; Singh and Laxmi, 2015; Wang et al., 2019). The components are ABA, PYR, PP2C, SnRK2, ABF, DREB2A, ABRE, and DRE (Figure 1).

We included other components necessary to connect each core component functionally to make a dynamic model. Previous studies have determined that the PP2C phosphatases dephosphorylate phosphorylated ABFs (Antoni et al., 2012; Lynch et al., 2012). The components were hence included in the model. In addition, another study identified that SnRK2s are enzymatically phosphorylated by MAP3Ks, RAF-like kinases, although the regulation of MAP3Ks by ABA has not yet been revealed (Katsuta et al., 2020; Lin et al., 2020; Takahashi et al., 2020). It is also known that BIN2 (BRASSINOSTEROID INSENSITIVE 2 kinase) phosphorylates SnRK2s, although its regulation by ABA is unknown (Cai et al., 2014). To this end, we added components that phosphorylate SnRK2s independently from the ABA regulation (presented as MAP3K in the model). DREB2A is subjected to 26 s proteasome proteolysis in a normal condition, but stress conditions block the proteolysis of DREB2A through a yet unknown mechanism (Qin et al., 2008). We added two interaction reactions to implement the finding in the model. One is between DREB2A and 26 s proteasome, the complex of which leads to degradation of DREB2A. The other is between 26 s proteasome and ABA, a complex which deactivates the degradation. We also included the feedback regulation in which the expression of PP2C, ABF, and DREB2A genes is upregulated by the ABRE promoter activity (Kim et al., 2011; Wang et al., 2019). In the dynamic model, a set of 33 variables and 63 parameters representing biochemical reactions of each component were constructed based on the law of mass action. In the model, a single protein in a homologous protein family that redundantly function in the cells regulates the system. Values of parameters in the equations were obtained from the literature (Table 1). The equations, initial conditions (concentrations), and parameter values were compiled and analyzed numerically with default settings using MATLAB SimBiology (MathWorks) (Supplementary File S1).

**Table 1:** Curated values from literature and the values chosen as parameters for the model.

Description	Reference	Value found in the literature	Parameter name in the model	Value used in the model	Fixed in the model*
*ABA and PYR1, PYL1, PYL2 binding	Dupeux et al., 2011	*K*_D_ = 52, 59, 97 μM	kf1 kr1	1,000 μM^−1^ s^−1^ 69,000 s^−1^	✓
*HAB1 and SnRK2.2, 3, 6 binding	Soon et al., 2012	IC_50_ 2 μM–8 μM	kf2 kr2	1,000 μM^−1^ s^−1^ 0.1 s^−1^	✓
*ABI1 and SnRK2.6-P binding	Xie et al., 2012	*K*_M_ = 0.097 μM	kf3 kr3	1,000 μM^−1^ s^−1^ 97 S^−1^	✓
SnRK2 and MAP3K binding	Ghose, 2019	*K*_M_ = 23 μM	kf4 kr4	1,000 μM^−1^ s^−1^ 23,000 s^−1^	✓
SnRk2.6-P and ABF-2 binding	Xie et al., 2012	*K*_M_ = 19.3 μM	kf5 kr5	1,000 μM^−1^ s^−1^ 19,300 s^−1^	✓
ABA interaction with 26S proteasome	Assumed		kf6 kr6	1,000 μM^−1^ s-1 50 s^−1^	✓
*PYR1.ABA and HAB1 binding	Dupeux et al., 2011	*K*_D_ = 30 nM	kf7 kr7	1,000 μM^−1^ s^−1^ 30 s^−1^	✓
*PYR1.ABA and HAB1.SnRK2 binding	Dupeux et al., 2011	*K*_D_ = 30 nM	kf8 kr8	1,000 μM^−1^ s-1 30 s^−1^	✓
ABF-P and PP2C binding	Pan et al., 2015	*K*_M_ = 11.15 μM	kf9 kr9	1,000 μM^−1^ s-1 11,150 s^−1^	✓
ABF-P and ABRE binding	Geertz et al., 2012	*K*_D_ of DNA-protein binding 2 nM–2 μM	kf10 kr10	1,000 μM^−1^ s-1 2 s^−1^	✓
DREB2A and DRE binding	Geertz et al., 2012	*K*_D_ of DNA-protein binding 2 nM–2 μM	kf11 kr11	1,000 μM^−1^ s-1 2 s^−1^	✓
ABF-P and ABRE binding	Geertz et al., 2012	*K*_D_ of DNA-protein binding 2 nM–2 μM	kf12 kr12	1,000 μM^−1^ s-1 2 s^−1^	✓
DREB2A and DRE binding	Geertz et al., 2012	*K*_D_ of DNA-protein binding 2 nM–2 μM	kf13 kr13	1,000 μM^−1^ s-1 2 s^−1^	✓
ABF-P and ABRE binding	Geertz et al., 2012	*K*_D_ of DNA-protein binding 2 nM–2 μM	kf14 kr14	1,000 μM^−1^ s-1 2 s^−1^	✓
Phosphorylation of SnRK2	Ghose, 2019	$k_{\mathrm{cat}}$ = 14 s^−1^	kf15	14 s^−1^	✓
DREB2A interaction with 26S proteasome	Assumed		kf16	5 μM^−1^ s-1	✓
Dephosphorylation of ABF-P by PP2C	Pan et al., 2015	$k_{\mathrm{cat}}$ = 1.04 s^−1^	kf17	1.04 s^−1^	✓
Release of SnRK2 from ABA.PYR.PP2C.SnRK2 complex.	Bar-Even et al., 2011	Average $k_{\mathrm{cat}}$ of enzyme reaction 10 s^−1^	kf18	10 s^−1^	✓
*Dephosphorylation of SnRK2.6-P	Xie et al., 2012	$k_{\mathrm{cat}}$ = 0.924 s^−1^	kf19	0.924 s^−1^	✓
*Phosphorylation of ABF-2 by SnRK2.6-P	Xie et al., 2012	$k_{\mathrm{cat}}$ = 0.04 s^−1^	kf20	0.04 s^−1^	✓
Transcription of ABRE genes	Hausser et al., 2019	<translation rate	kf26	10 h^−1^	
Transcription of *RD29A* gene	Hausser et al., 2019	<translation rate	kf27	10 h^−1^	
Translation of ABRE genes	Hausser et al., 2019	<10,000 h^−1^	kf28	200 h^−1^	
Transcription of constitutively expressed genes	Hausser et al., 2019	<translation rate	kf29	1 h^−1^	✓
Translation of constitutively expressed genes	Hausser et al., 2019	<10,000 h^−1^	Kf30	4.5 h^−1^	✓
Degradation of protein	Hausser et al., 2019	Protein decay rate in Hela cells 0.05 h^−1^	kf21-kf25, kf31-kf 45, and kf49	0.05 h^−1^	✓
Degradation of mRNA	Hausser et al., 2019	mRNA degradation in HEK293 cells 0.06 h^−1^	kf46, kf47, kf48	0.06 h^−1^	✓

Each reaction in the model was shown with the respective parameter and the source from which the value was obtained. (✓) = Fixed in the model: ✓ indicates the value used in the model was not altered during model optimization.

(*) = Parameters are derived from plant proteins and respective homologous proteins are indicated.

In the model, we assumed:

- ABA signal transduction occurs through the system composed of ABA, PYR, PP2C, SnRK2, ABF, DREB2A, ABRE, and DRE, in which *RD29A* is expressed.
- Enzymatic reactions follow Michaelis–Menten kinetics.
- All molecules freely diffuse in the cell. This is not true for all components, but the assumption is necessary for modeling.
- The cell volume is 50 μm^3^.
- The Michaelis constant is $K_{M}$ = $\frac{k_{r}+k_{\mathrm{cat}}}{k_{f}}$ , where $k_{r}$ is the dissociation rate constant, $k_{\mathrm{cat}}$ is the catalytic rate constant, and $k_{f}$ is the association rate constant.
- A molecule associates with another molecule at a rate constant of $k_{f}$ = 1,000 μM^−1^ s^−1^ (Milo and Phillips, 2015).
- Proteins are generated by reactions of gene expression and protein translation, then subject to degradation.
- The protein concentration in a cell remains at 0.1 μM at a steady state without ABA activation and feedback regulation.
- A gene (mRNA) is expressed from a pair of gene loci with a constitutively active promoter and then subjected to degradation.
- Genes with feedback regulation (ABF, PP2C, DREB2A) have ABREs and a constitutively active promoter. Hence, upon binding the activated ABF, expression is increased compared to the level of constitutive expression.
- The binding of ABF and DREB2A to ABRE and DRE, respectively, is required for *RD29A* expression.
- Initial values of variables (components) are 0 (zero) except for the gene in the genome.

The model was first to run for 300 equivalent hours in numerical analysis with the variable ABA (representing intracellular ABA) set at 0 μM. This allows the system to reach a quasi-steady state. After the 300 equivalent hours, the variable ABA was set to 100 μM. Changes in all variables in the model from the quasi-steady state were then monitored for another 300 equivalent hours. This report presents the time when the variable ABA is changed to time zero.

### Optimization of parameters, validation of the model, and analyzing identifiability of model parameters

We approximately curve fit model output to experimental data to optimize selected model parameters. We focused on changes in the variable *RD29A*, representing accumulated mRNA expressed. Three parameters, 1. transcription of feedback ABRE containing genes, 2. transcription of *RD29A,* 3. translation of PP2C, ABF, and DREB2A affected by feedback, were manually changed to obtain a qualitatively good fit to experimental data. The remaining model parameters were unchanged (fixed). To validate the model, we quantitatively evaluated changes of the variable *RD29A*. Fold changes calculated by the model were compared to previously published data or newly obtained in this study. Our new experiments used the transgenic *Arabidopsis thaliana* plant (Supplementary Methods). The transgenic plant carries the *RD29A::LUC* gene expression cassette that drives the expression of luciferase (LUC) from the *RD29A* promoter in the genome containing ABRE and DRE *cis-*elements (Zhan et al., 2012). Because the half-life of luciferase is shorter than that of the mRNA, the activity of luciferase that is detected as the emission of light (luminescence) can be used to track the accumulation of the mRNA in near real-time. To analyze the identifiability of the variable *RD29A* dynamics, we conducted a sensitivity analysis using Calculate Sensitivity in Model Analyzer in SimBiology with default settings.

## Results

### Over 50% of the genes in the ABA gene regulatory network, including *RD29A*, contain a single ABRE in the gene body

**Figure 2:**
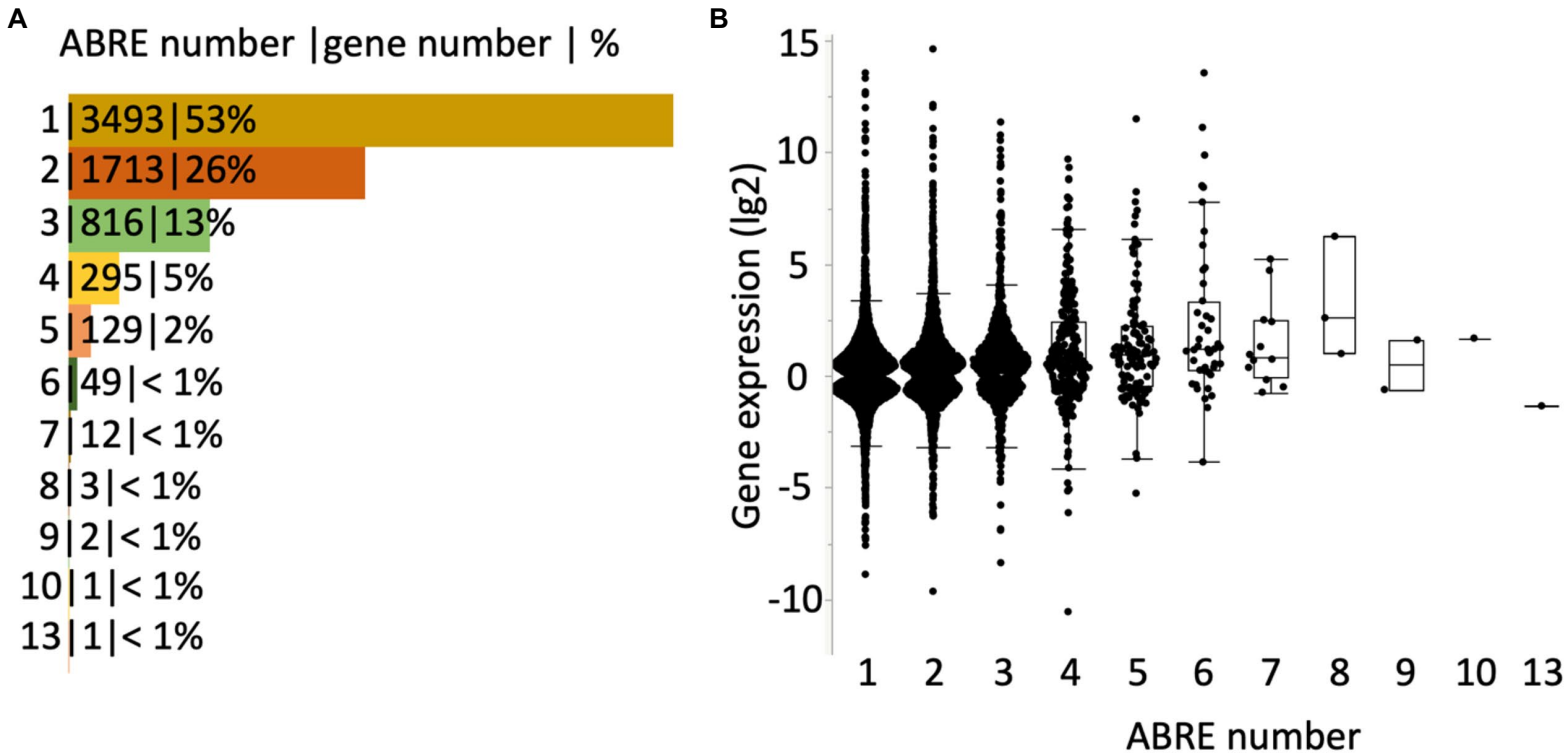
Genes regulated through ABRE-ABF binding in Arabidopsis. **(A)** Genes in the ABA GRN (Sun et al., 2022) are categorized by the number of ABRE in their gene body. The number of genes belonging to each category and percentage in the ABA GRN is shown with a separate marker “|.” The bars in the figure indicate the percentages. **(B)** Box plots of genes in the ABA GRN. X-axis variables are the number of ABRE in a gene. Y-axis variables are changes in gene expression (lg2) by ABA in root tissue (3 h after the ABA treatment). Black dots indicate points of individual genes. All genes in the ABA GRN are listed, together with gene expression in root and shoot tissues at different time points, in Supplementary Table S1.

A most recent and comprehensive study conducted by Sun et al. identified genes that are differentially expressed by ABA and the number of ABRE in the genes in *Arabidopsis* (Sun et al., 2022). A set of genes whose expression is significantly altered by 10 μM ABA through binding of activated ABF on ABRE in the root or shoot tissue in 7 days old seedlings were defined as an ABA GRN (gene regulatory network) (Sun et al., 2022). We found that 53% of the GRN carry a single ABRE within the gene body (Figure 2A and Supplementary Table S1). The range of a fold-change in the gene expression in the genes carrying a single ABRE in their gene body is as wide as those carrying multiple ABREs in their gene body (Figure 2B and Supplementary Table S1). This suggests that the genes carrying a single ABRE are the major contributors to the ABA GRN. *RD29A*, one of the most studied genes as the ABA-induced gene marker, also carries a single ABRE in the gene body (Sun et al., 2022).

### Parameter values of the *RD29A* gene expression were obtained by literature curation

To understand the alteration of gene expression kinetics by the single ABRE, a dynamic model of the *RD29A* gene expression was constructed with ordinary differential equations. We curated previously published data to define parameters in the model of the ABA signaling pathway that activates the ABF, resulting in the activation of the gene promoter containing the ABRE and DRE. The summary of our curation is shown below (Table 1).

**Figure 3:**
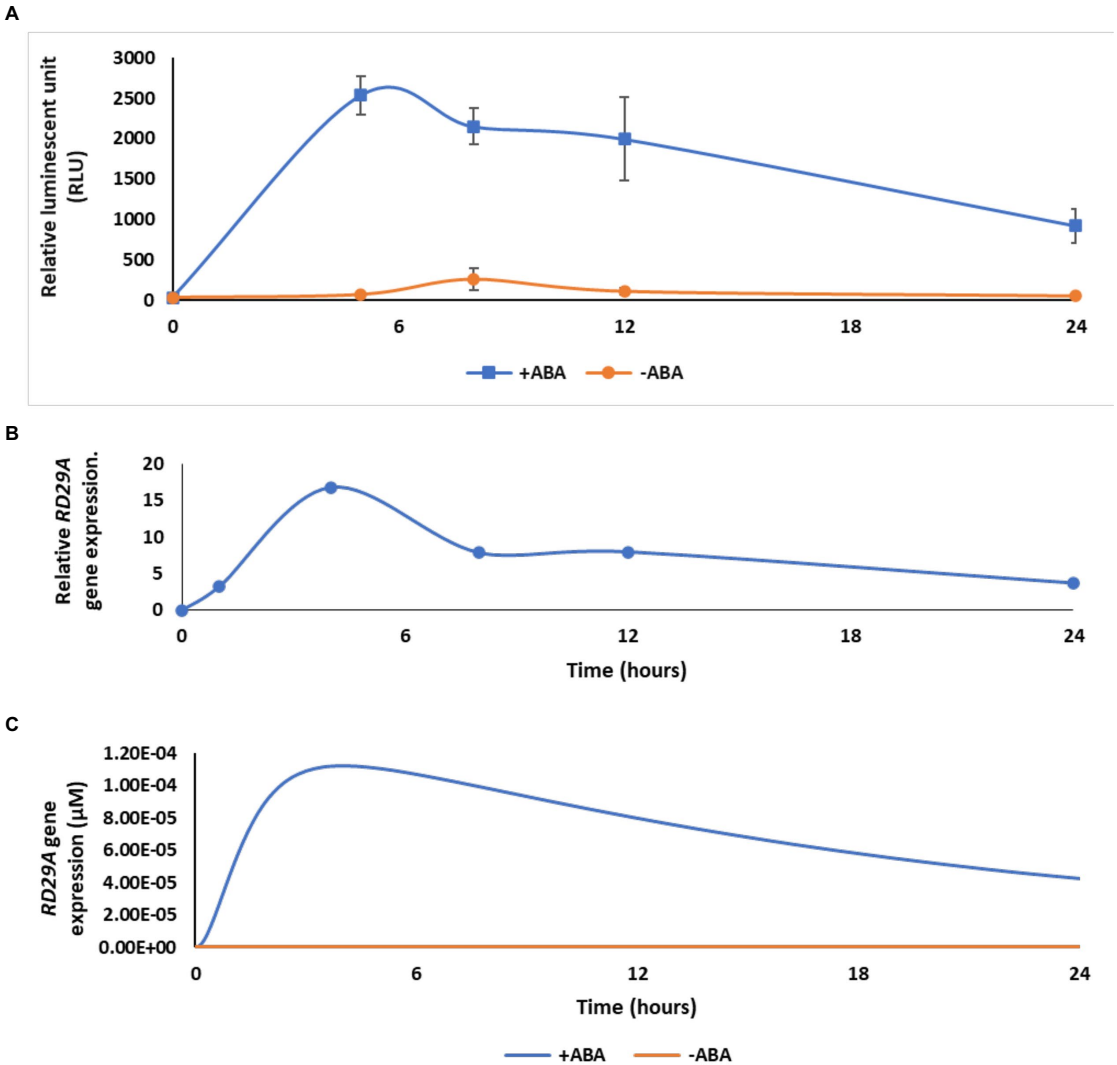
The dynamic model agrees with ABA-induced gene expression in real plants after optimization. **(A)** Kinetics of luciferase activity in the *RD29A::LUC* plant after exposure to 200 μM ABA (+ABA) or DMSO for control (-ABA). The graph shows the mean of three independent experiments. Error bars represent standard error from the mean. **(B)** Kinetics of *RD29A* gene accumulation in the previously published data with 10 μM ABA in Arabidopsis (Song et al., 2016). **(C)** Model output with showing transient expression after optimization of parameters kf26, kf27, and kf28.

While parameter values for protein–protein interactions and enzymatic reactions were characterized *in vitro* studies using recombinant proteins, no studies related to parameter values of DNA-protein binding, gene expression, protein translation, and degradation were found for the ABA signaling pathway. To this end, we implemented parameter values from studies using non-plant eukaryotic organisms. These parameters had a wide range to select from 1. equilibrium dissociation constant between the transcription factors and respective *cis-*elements, (from 2 nM to 2 μM) (Geertz et al., 2012), 2. translation rate of protein from mRNA expressed (less than 10,000 h^−1^) (Hausser et al., 2019), 3. transcription rates (slower than the translation rate) (Hausser et al., 2019). We selected the translation and transcription rates for genes at 4.5 h^−1^ and 1 h^−1^, respectively, and 2 nM for transcription factor-*cis-*element binding. This is because the average rate of gene transcription in multicellular eukaryotes is 1 h^−1^ (Hausser et al., 2019), while the average concentration of proteins involved in signal transduction is 0.1 μM (Milo and Phillips, 2015). Setting translation rate at 4.5 h^−1^ and transcription rate at 1 h^−1^ makes the concentration of a protein at a quasi-steady state to 0.1 μM without ABA and feedback regulation in our model. The affinity of transcription factor-*cis-*element binding was set at 2 nM to fit the kinetics of the variable *RD29A* with actual gene expression (Figure 3). Protein degradation was set at 0.05 h^−1^ (Hausser et al., 2019). The equilibrium dissociation constant between SnRK2 (non-phosphorylated SnRK2) and PP2C was set at 100 pM, representing complete inhibition of SnRK2 kinase activity by PP2C at an equal molar concentration (Soon et al., 2012).

### The rates of transcription and translation in *RD29A* and the feedbacked genes, *ABF*s, *PP2C*s, and *DREB2A*, were optimized in the model to capture observed dynamics in experimental data

To understand the connectivity of the components, we compared the kinetics of gene expression in the model and experimental data in actual plants. We compared the simulation data of the variable *RD29A*, to two independent data sets that were experimentally obtained using actual plants. One set of data was obtained by our new experiments using transgenic *Arabidopsis thaliana* carrying the *RD29A::LUC* (Zhan et al., 2012). The other set was obtained from previously published data that show a change in the *RD29A* gene expressed in *Arabidopsis thaliana* (Song et al., 2016). Kinetics of the gene expression in the plants and the variable *RD29A* were compared within the first 24 h (Figure 3).

Experimental data from the transgenic *RD29A::LUC* plants showed transient activation of its promoter with an initial increase and then a decrease after 5 h (Figure 3A). Similar transient expressions of the *RD29A* gene were observed in non-transgenic *Arabidopsis* plants, (Song et al., 2016) (Figure 3B). Despite the *RD29A* transient expression pattern being consistently observed in both transgenic and wild-type plants, the mechanism that explains the transient expression was unknown. Therefore, we investigated the mechanism using the dynamic model we constructed. When we simulated the kinetics of the variable *RD29A*, the kinetics were logarithmic upon adding ABA. We therefore optimized the parameters so that the model’s kinetics in the gene expression qualitatively agree with that in actual plants (Figure 3C). We altered three parameters, the feedback transcription rate constant of the ABRE promoter (parameter kf26), the transcription rate constant of *RD29A* with both DRE and ABRE elements (parameter kf27), and the feedback translation rate constants of ABF, PP2C, and DREB2A (parameter kf28). These three parameters had not been determined previously, and studies in other eukaryotic cells indicate wide ranges of reasonable values (Table 1). Hence, we manually altered the values within the ranges of a previous biological study (Hausser et al., 2019) so that the kinetics of the variable *RD29A* resembles the actual plant data. Changes on these parameters most affected the aspect of transient increase of the variable *RD29A*. The values 5 < kf26 < 10 h^−1^, 5 < kf27 < 10 h^−1^, and 150 < kf28 < 250 h^−1^ recapitulated the trend of gene expression (Figure 3).

### Approximation of the model was validated by determining model responses to different doses of ABA or a set of gene null-mutations

**Figure 4:**
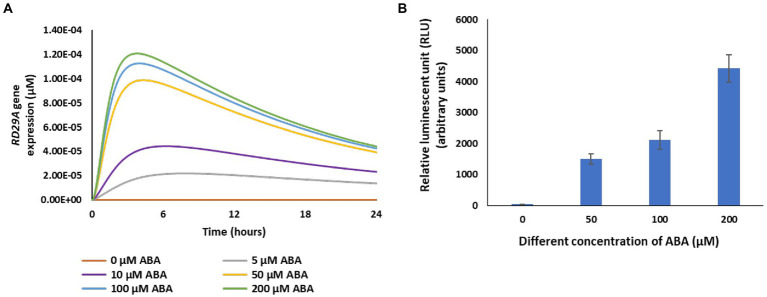
RD29A expression increases with a function of ABA concentration in the model as it is observed in actual plants. **(A)** Model output of the variable *RD29A* with different values of the variable ABA. **(B)** The relative luminescence unit in 25-day-old *RD29A::LUC* plants was determined at 5 h after spraying different concentrations of ABA. The bars represent the mean relative luminescence of three replicates, with error bars representing standard error from the mean (15 seedlings).

To validate the model, we first compared the ABA-dose-dependent response in actual plants to the dynamics of the variable *RD29A* (Figure 4). In the model, changes of the variable *RD29A* increased in an ABA-dose-dependent manner from 0 to 200 μM (Figure 4A). With the *RD29A::LUC* transgenic plants, changes in luminescence increased in an ABA-dose-dependent way in the range from 0 to 200 μM (Figure 4B). The results would depend very much on time after treatment with ABA. Hence, we sampled the actual plants 5 h after exposure to ABA when the largest dose–response would be expected. The comparison of the outcomes suggested that the model is approximated to actual plants concerning ABA sensitivity. However, the response in the model seems to have narrower sensitivity against the ABA concentration (i.e., from 0 to 50 μM) compared to that in the actual plants (i.e., from 0 to 200 μM) (Figure 4B) (Gampala et al., 2001; Lee et al., 2016).

We also validated changes of the variable *RD29A* in gene-knockout simulations. Namely, we simulated the expression *RD29A* gene in gene null-mutations of *pyr*, *pp2c*, *snrk2*, and *abf*, which were previously studied (Fujita et al., 2009; Rubio et al., 2009; Nishimura et al., 2010; Yoshida et al., 2015). In these studies, different stages of seedlings were treated differently with ABA. Hence, we evaluate the expression of *RD29A* qualitatively (increased or decreased) but not quantitatively (i.e., comparing changes in the expression kinetics). We simulated knockout mutations by setting the translation rate constant (kf30) to zero for the variable PYR, PP2C, SnRK2, and ABF. In addition, we also set the translation rates of the feedback regulations kf28 to zero for ABF and PP2C, respectively. The mimicked null-mutant in *pyr*, *snrk2*, and *abf*, all showed reduced levels of the variable *RD29A*, while the mimicked null-mutant in *pp2c* showed elevated levels (Table 2).

**Table 2:** Mutant simulations in the model show qualitative similarity to actual mutant plants, concerning the *RD29A* expression.

A variable set to 0 in the model	Highest *RD29A* concentration in the model (μM)	Knockout genes in actual plants	*RD29A* gene expression in the knockout plants exposed to ABA	Reference
None	1.13E-4	None (wild type)	transient	Song et al., 2016
PPC2	6.24E-3	*pp2ca/hai1*	constitutive and high	Antoni et al., 2012
PYR	2.57E-6	*pyr1/pyl1/pyl2/pyl4*	impaired	Park et al., 2009
SnRK2	0	*snrk2.2/ snrk2.3 snrk2.6*	impaired	Thalmann et al., 2016
ABF	0	*areb1/areb2/abf3*	impaired	Thalmann et al., 2016

Mutant simulations were made on the model with the variable ABA set at 100 μM. Highest concentration of the variable *RD29A* at each of the simulations was recorded. Relative expression of the *RD29A* gene in actual plants was curated from previously published literature.

Experimental data in actual plants shows that *pyr* null-mutants are impaired in ABA-induced gene expression (Park et al., 2009; Nishimura et al., 2010; Gonzalez-Guzman et al., 2012). Similarly, experimental data on *snrk2.2/ snrk2.3/ snrk2.6* triple knockout mutants showed that the expression of ABA-induced genes was impaired (Fujii and Zhu, 2009; Fujita et al., 2009; Thalmann et al., 2016). Triple *areb/abf* mutants were found to have reduced ABA-induced gene expression (Yoshida et al., 2015; Thalmann et al., 2016). On the other hand, null mutants of *pp2cs* in actual plants show a higher and constitutive ABA response (Rubio et al., 2009; Antoni et al., 2012). Based on the two validations described above, we concluded that the model constructed, and parameters implemented in the model are approximated to actual plants.

### The dynamic model predicts that a single ABRE makes the *RD29A* expression transient through the PP2C feedback loop during ABA exposure

**Figure 5:**
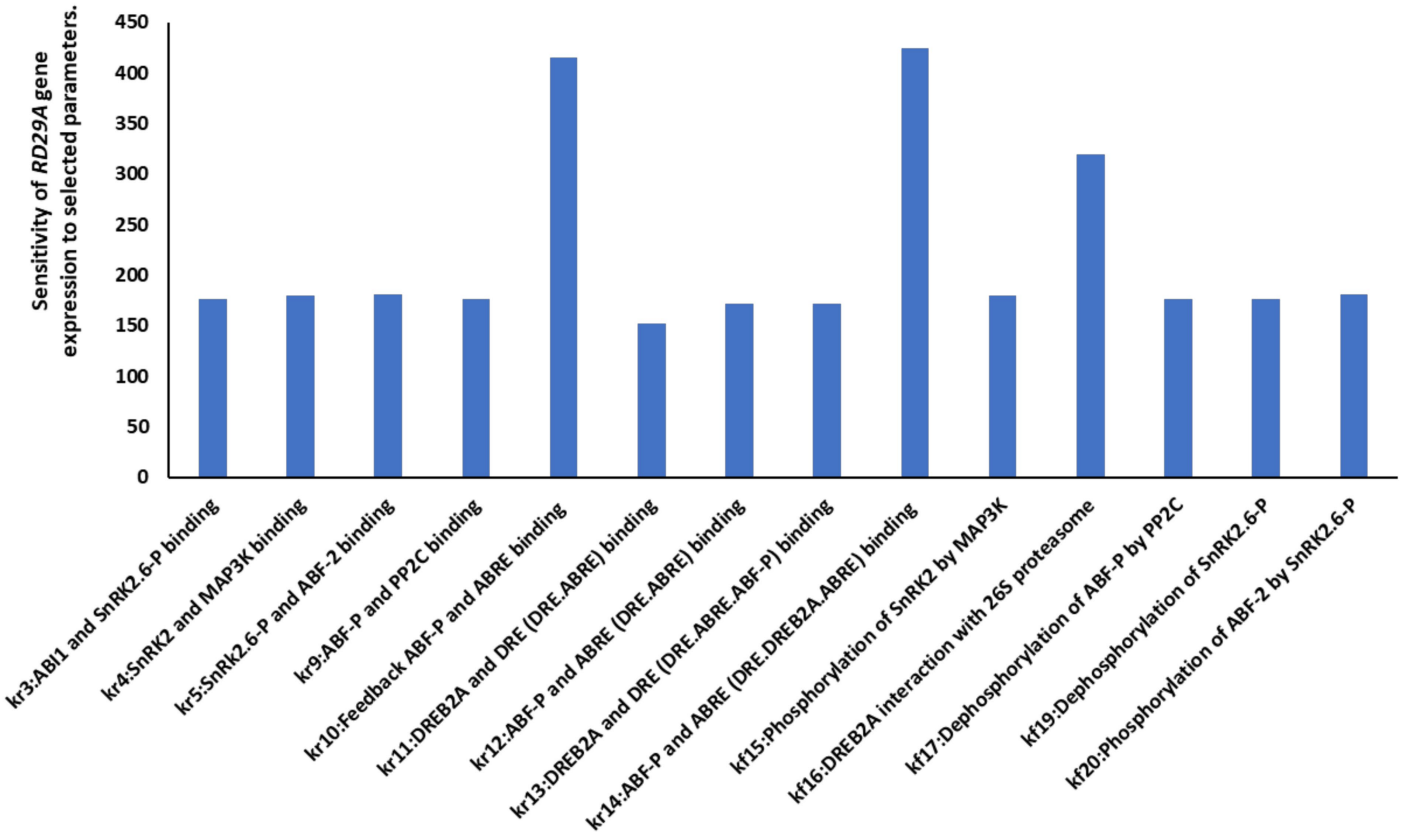
Sensitivity analysis of different parameters on *RD29A* gene expression. A sensitivity analysis conducted against the variable *RD29A* gene determined that the parameters for ABF-P-ABRE binding are most sensitive to the kinetics of *RD29A* gene expression. The y-axis shows the level of influence of the respective parameter on the output or *RD29A* gene expression. The higher the value, the higher the effect of the parameter on the gene expression.

To understand which parameters are sensitive for the *RD29A* gene expression, we conducted a sensitivity analysis on fourteen parameters that determine protein binding, enzymatic activity, and protein degradation of the key compartments were selected (Figure 5**)**. The analysis showed that the parameter related to the ABF-P binding to ABRE for the feedbacked regulation genes (ABF, PP2C, ABF, DREB2A) (kr10) and the parameter related to the ABF-P biding to ABRE for the *RD29A* expression (kr14) were the most sensitive. Parameters associated with DREB2A binding of the DRE were not significantly sensitive as those related to ABF-P binding to ABRE (Figure 5). However, the binding affinity of DREB2A-DRE certainly affected the expression levels of the *RD29A* mRNA (Supplementary Figure S1). Interaction of DREB2A and 26 s proteasome (kr16) was most sensitive after those related to ABF-P binding to ABRE.

**Figure 6:**
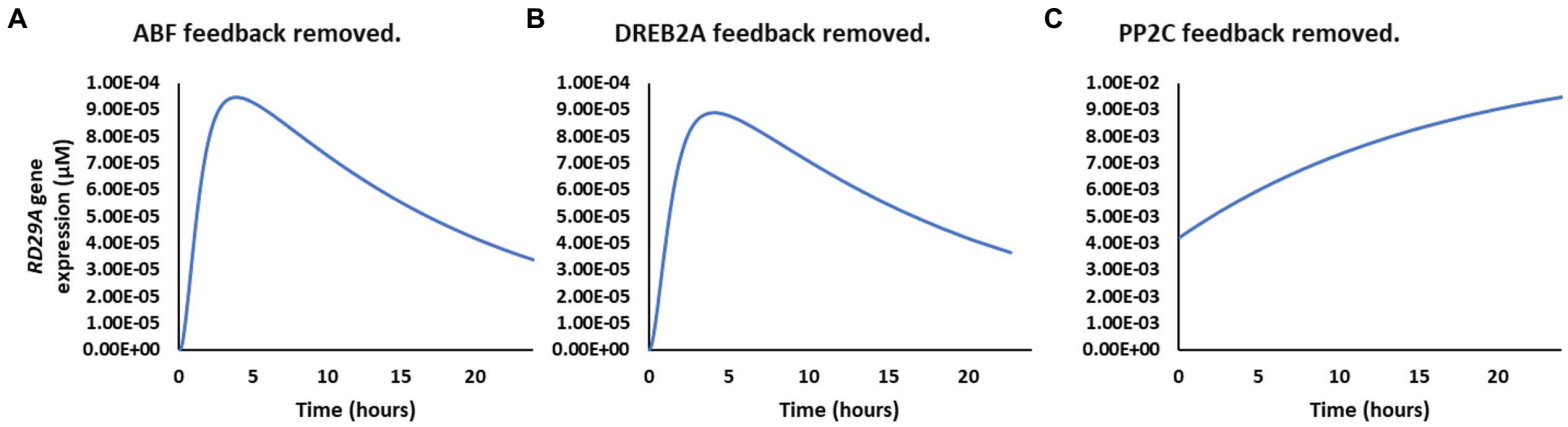
Effect of the different feedback loops on *RD29A* gene expression dynamics. **(A)** Without the ABF feedback loop. **(B)** Without the DREB2A feedback loop. **(C)** Without the PP2C feedback loop.

Because the parameter representing feedback loops of the ABA signaling pathway was identified as the most sensitive for the *RD29A* expression, we individually examined the effect of the ABF, DREB2A, and PP2C feedback loops. On removal of the feedback loop on *ABF* or *DREB2A*, the expression dynamics did not change. Still removing the PP2C feedback loop created logarithmic gene expression (Figure 6). Also, the concentration of *RD29A* mRNA increased about 100-fold higher without the *PP2C* feedback loop. These indicate that the feedback loop of the *PP2C* gene expression makes the *RD29A* gene expression transient and reduces the dynamic range.

## Discussion

The ABRE is the main element involved in ABA-mediated gene expression. However, previous studies have shown that a single ABRE copy is not sufficient for gene expression, and other elements like DRE, CE1, or CE3 are required as coupling elements. (Shen et al., 1996; Abe et al., 1997; Hobo et al., 1999; Uno et al., 2000; Zhu, 2002). Yet, the most comprehensive genome-wide study indicated that genes carrying a single ABRE are the major genes in the ABA GRN (Sun et al., 2022) (Figure 2).

The role of the single ABRE on the gene expression dynamics in the ABA GRN was unknown (Narusaka et al., 2003; Sun et al., 2022). Here, we presented a model of the ABA signaling pathway describing the expression dynamics of *RD29A* under the control of the ABRE and DRE (Figure 3). The model was built with fixed parameter values of protein–protein interactions and enzymatic kinetics obtained by *in vitro* experiments from the literature. The model was further validated by comparing *RD29A* gene expression from the model’s output to actual plant data (Figure 4 and Table 2). The model suggests that a single ABRE controls the transient nature of the gene expression through the feedback loop of the *PP2C* gene expression (Figure 6). In addition, the model suggests that PP2C largely suppressed the expression level of *RD29A* (Figure 6). On the other hand, the feedback loop of DREB2A that binds to DRE does not affect the kinetics of the *RD29A* expression (Figure 6), despite a fact that DRE also regulates the level of the ABA-dependent RD29A gene expression (Supplementary Figure S1). Based on the model analyses, we hypothesize that a single ABRE regulates the time scale and dynamic range of the expression in the ABA GRN although the single ABRE itself is not sufficient to drive the expression.

In this study, a direct quantitative comparison between the model output and actual data was not conducted because we modeled the system as a single cell, whereas the actual data obtained from the multicellular system. Furthermore, each homologous protein that redundantly functions in the signaling pathway may have a different parameter. For instance, the homologous protein family of 14 PYR and 9 PP2Cs were shown to have different affinities in the ABA-responsive gene expression (Tischer et al., 2017). Accordingly, our model cannot simulate the plant response exactly. For instance, when an ABA-concentration-dependent response of the ABRE promoter was determined, the response range was narrower in the model than in existing plants (Figure 4). Optimization of parameter values fixed in this study may be required to improve model performance.

Nevertheless, our model successfully builds off existing work to represent the relationship between the ABA signaling pathway and the gene expression regulation by a single ABRE. The model construed in this study underpins the importance of a single ABRE in the ABA GRN. Revealing the interaction between the single ABRE with other *cis*-elements in the regulatory region of each gene would be the next frontier for understanding the ABA GRN.

## Data availability statement

The datasets presented in this study can be found in online repositories. The names of the repository/repositories and accession number(s) can be found at: https://www.ncbi.nlm.nih.gov/genbank/, AY142623, BT008860, BT015409, BT025246, BT002082, AY081538, BT026443, AY081467, and AY091298.

## Author contributions

NK: conceptualization, methodology, and funding acquisition. RN and RD: validation. RN: experiments. RN and NK: formal analysis and writing—original draft preparation. RN, RD, and NK: writing—review and editing. All authors contributed to the article and approved the submitted version.

## Funding

This study is partly supported by Economic Development Assistantships from Louisiana State.

## Conflict of interest

The authors declare that the research was conducted in the absence of any commercial or financial relationships that could be construed as a potential conflict of interest.

## Publisher’s note

All claims expressed in this article are solely those of the authors and do not necessarily represent those of their affiliated organizations, or those of the publisher, the editors and the reviewers. Any product that may be evaluated in this article, or claim that may be made by its manufacturer, is not guaranteed or endorsed by the publisher.
