# Supplementary material for: Dynamic modeling of ABA-dependent expression of the Arabidopsis RD29A gene
Source: Front Plant Sci. 2022 Aug 26;13:928718. doi: 10.3389/fpls.2022.928718 (PMC9458874; doi:10.3389/fpls.2022.928718)
Supplement: Supplementary file 3 [file Data_Sheet_2.pdf]

Supplementary methods

Transgenic plant and growth conditions

Transgenic *Arabidopsis thaliana* seeds (CS67900) that carry an *RD29A::LUC* gene expression cassette in the genome were obtained from Arabidopsis Biological Resource Center (ABRC). They were sterilized in 70% ethanol for 1 minute, 50% bleach, and 0.05% triton for 10 minutes. The seeds were rinsed 6 times with sterilized distilled water and then plated on ½ Murashige-Skoog (MS) medium (Murashige & Skoog, 1962) containing 0.8% agar. They were then stratified at 4 ^{o} C for three days, then placed in a growth chamber under a growth cycle of 16 hours day 8 hours dark with a light set at 100 µmol m^{-2} s^{-1} and 22 ^{o} C.

Long-term luminescence assay with transgenic *Arabidopsis thaliana*

Twenty-five-day-old transgenic *Arabidopsis thaliana* plants on an agar plate were sprayed with 200 µM ABA and for control with the same concentration (v/v) of DMSO for different periods (0, 5, 8, 12, and 24 hours). Fifteen seedlings were harvested at each time point, frozen immediately in liquid N_2, and kept at –80°C until analysis. For analysis, frozen plants were ground using a mortar and pestle to form a powder. The powder was mixed with passive lysis buffer (Promega: Cat. #E1941). The mix was then vortexed and centrifuged at 14,000rpm for 10 minutes. The supernatant (40 µL) was placed in a well on a 96 well plate (Thermo Fischer cat# 267350) and mixed with 100 µL luciferase assay substrate (Promega: Cat. #E151A). Relative Luminescence Unit (RLU) was detected using a Veritas ™ microplate luminometer. Three RLU readings were made for each well and then averaged as data.
