# Supplementary material for: Dynamic modeling of ABA-dependent expression of the Arabidopsis RD29A gene
Source: Front Plant Sci. 2022 Aug 26;13:928718. doi: 10.3389/fpls.2022.928718 (PMC9458874; doi:10.3389/fpls.2022.928718)
Supplement: Supplementary file 4 [file Image_1.pdf]

Supplemental Figures

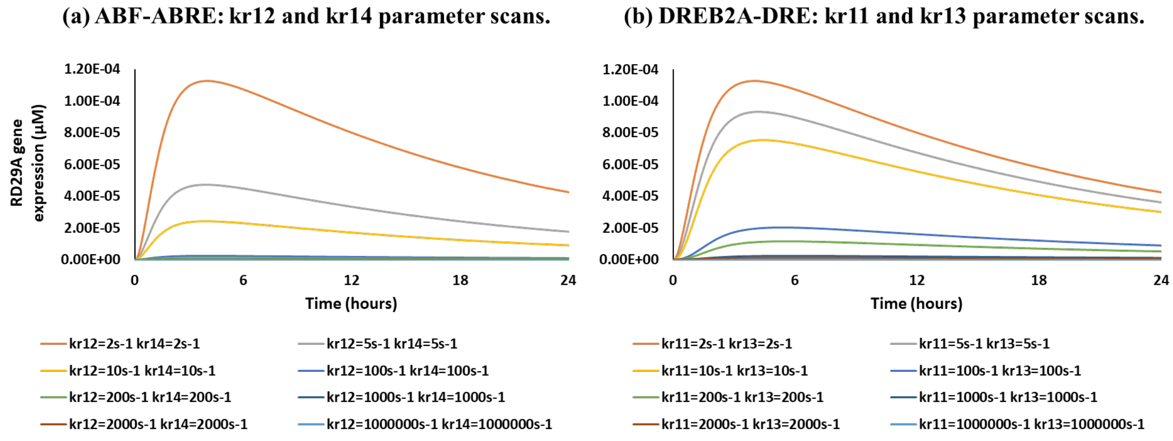

Figure S1. Changes in the *RD29A* gene expression with a function of binding affinity. The *RD29A* gene expression was stimulated with the alternated binding affinities, 2, 5, 10, 100, 200, 1000, 2000, and 10^6 µM in (**a**) ABF-ABRE interaction (parameters kr12 and kr14) or (**b**) DREB2A-DRE interaction (parameters kr11 and kr13).
